# Synergetic Effect of Superabsorbent Polymer and CaO-Based Expansive Agent on Mitigating Autogenous Shrinkage of UHPC Matrix

**DOI:** 10.3390/ma16072814

**Published:** 2023-03-31

**Authors:** Yang Chen, Rong Xian, Jiawei Wang, Zhangli Hu, Wenbin Wang

**Affiliations:** 1 School of Materials Science and Engineering, Southeast University, Nanjing 210096, China; remember_chenyang@163.com (Y.C.);; 2 Guangdong Bay Area Transportation Construction Investment Co., Ltd., Guangzhou 510030, China; 3 Jiangsu Sobute New Material Co., Ltd., Nanjing 211103, China

**Keywords:** ultra-high-performance concrete, superabsorbent polymer, CaO-based expansive agent, synergetic effect, autogenous shrinkage

## Abstract

The hybrid use of a superabsorbent polymer (SAP) and expansive agent (EA) is beneficial for mitigating the autogenous shrinkage of ultra-high-performance concrete (UHPC) without compromising strength. However, the unclear mechanisms behind the synergetic effect of the two materials may hinder the more effective applications of this method. This study clarifies the interactions between SAP and CaO-based EA (CEA) in a UHPC matrix by quantifying the content and distribution of water and hydration products, underlining their influence on the strength and autogenous shrinkage evolution. The high strength of 135 MPa can be achieved in systems with a reasonable combination (S1E1, 0.1 wt%SAP, and 1 wt%CEA), and after 7 days, a 24% reduction in shrinkage was found in the same system, which is more effective than the use SAP or CEA alone at the same dose. The mitigating effect on the autogenous shrinkage of a UHPC matrix with hybrid materials at different stages depends on the competition between the water retention for self-desiccation and portlandite formation. With the continuing formation of hydration products, the microporosity of UHPC matrix under internal curing conditions at 28 d is considerably reduced, resulting in a more compact microstructure. This study also finds a suppressed crystallization pressure of growing portlandite in the extra space provided by emptied SAP, which explains the lost expansion of CEA.

## 1. Introduction

Currently, advanced materials are widely used in the fields of construction machinery, aerospace, environmental protection, and engineering construction [1,2,3,4,5,6,7].

Among these advanced materials, UHPC is a revolutionary cementitious material that has progressively been employed in infrastructure development worldwide over the last three decades. In comparison to traditional concrete, this material has superior strength, ductility, and durability. These qualities are achieved by removing coarse aggregate, optimizing the mix design, applying steam curing, and incorporating steel fibers [8,9]. Due to its high cementitious content (more than 800 kg/m^3^) and low water–binder ratio (w/b < 0.20), the autogenous shrinkage of UHPC is much larger than that of ordinary concrete [10]. Autogenous shrinkage plays a major role in shrinkage (including autogenous shrinkage and dry shrinkage) in UHPC, which often results in cracking during construction and service periods. Cracking in concrete can cause a loss of strength and generate a penetrating path for air and water with harmful ions, leading to the deterioration of concrete [4]. Therefore, it is necessary to control the autogenous shrinkage of UHPC.

At present, the most prevalent shrinkage mitigation techniques for UHPC are the use of internal curing via a superabsorbent polymer (SAP) [11,12] and porous lightweight aggregate [13], an expansion agent [14], and a shrinkage-reducing agent (SRA) [15]. Two of the most commonly used expansive agents are calcium sulfoaluminate- and lime-based materials (CaO-based) (CEA), which react with water to form expansive products. After reacting with water to form boronite, the CaO-based expansion agent can increase by about 90% in volume [16,17]. Whatever type of expansion agent has been adopted, water is an essential reactant in an expansive reaction, highlighting the significance of the curing technique [18]. It is difficult for conventional curing techniques (such as water curing, sheet curing, and permeable formwork curing) to have a substantial influence on the curing of low water–binder ratio concrete via external moisture penetration. As an internal curing material, SAP delivers internal curing water and maintains internal relative humidity [19,20], which can be used as a source of hydration for the expansive agent. Indeed, the autogenous shrinkage reduction impact of CEA/SAP alone cannot fulfill the UHPC requirements for shrinkage reduction. Nowadays, the combination of SAP and growth agents is progressively becoming the mainstream option [21,22,23,24].

At different stages, CEA and SAP have synergistic effects on the mitigating autogenous shrinkage of UHPC. At early ages, SAP can supply sufficient water to minimize autogenous shrinkage and, at the same time, promote CEA expansion to further compensate for the high early shrinkage of UHPC [25,26]. SAP particles enhance the quick healing of fractures by themselves and the formation of a hydrated cement phase in native pores [22]. The addition of CEA can also promote the hydration of cement [23]. Nevertheless, the exact mechanism by which the SAP increases CEA hydration is still unclear. The role of humidity in the hydration process is also under debate. Therefore, it is important to investigate the distribution of water, the amount and distribution of expansion products, and the impact of pore structure on strength and autogenous shrinkage.

This article systematically investigates the influence of different dosages of SAP and CEA on the autogenous shrinkage and strength of a UHPC matrix. The internal curing impact of SAP on a UHPC matrix, in particular the internal humidity distribution, was investigated in depth. The mechanism of strength and autogenous shrinkage of a UHPC matrix under the hybrid use of SAP and CEA was determined, and a novel phenomenon of expansion failure caused by the combined use of SAP and CEA was discovered. Additionally, the thermogravimetric results verify that the hydration products of CEA are generated in a large amount in the mixed system. Mercury intrusion experiments and SEM confirmed that the CEA product did not provide sufficient expansion efficiency due to its ineffective expansion in SAP pores. The findings of this research lay the foundation for the engineering application of the combination of different shrinkage mitigation measures.

## 2. Materials and Methods

### 2.1. Raw Materials and Mix Design

**Figure 1:**
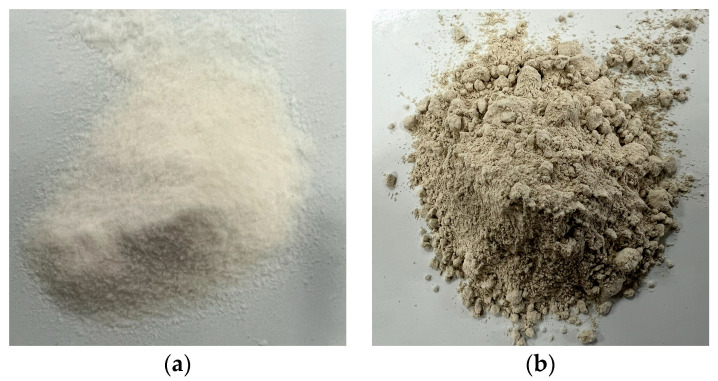
Raw materials. (**a**) SAP. (**b**) CEA.

Portland cement of P.II 52.5 Mpa was used. The ultra-fine mineral admixture was composed of ultra-fine slag, silica fume, and microspheres, etc., and is commercially available as SBT-HDC (V). It had a density of 2.45 g/cm^3^ and a specific surface area of 8500 m^2^/kg. Table 1 shows the chemical composition of cement and HDC (V). The CEA was provided by Jiangsu Sobute New Material Co., Ltd. (Nanjing, China) Calcium oxide was the primary expansion source of calcium expansion agent (CEA) used in this study, whereas sulfoaluminate was the secondary expansion source. Table 2 shows the CEA phase composition. The SAP was synthesized via the copolymerization of acrylic acid and acrylamide (1:1). The SAP particle size distribution is shown in Table 3. The SAP absorption was measured using the tea bag method. The SAP and CEA used in the experiment are shown in Figure 1.

The w/b of the reference (U0) was 0.175. CEA was added in two proportions, namely 1 wt% and 2 wt%, and the corresponding samples were named E1 and E2, respectively. SAP was added at 0.1 wt% (S1) and 0.2 wt% (S2), and additional water was introduced according to its water absorption capacity of 20 g/g [27]. The samples used in combination with SAP and CEA and their proportions are: S1E1 (0.1 wt% SAP, 1 wt% CEA, w/b = 0.195), S1E2 (0.1 wt% SAP, 2 wt% CEA, w/b = 0.195), S2E1 (0.2 wt% SAP, 1 wt% CEA, w/b = 0.215) and S2E2 (0.2 wt% SAP, 2 wt% CEA, w/b = 0.215).

A polycarboxylate-based superplasticizer (PCA) was used for all mixtures to maintain the workable fluidity of mortar with an extremely low water–cement ratio. Natural sand with fineness modulus of 2.7 and apparent density of 2641 kg/m^3^ was used as the fine aggregate. The mix design is shown in Table 4.

### 2.2. Compressive Strength

The Chinese Standard GB/T 17671-2021 [28] was referenced to test the compressive strength. For each sample, three 40 × 40 × 40 mm^3^ molds were filled with the mortar that had been mixed in a vacuum mixer. After 24 h, the samples were firstly demolded, then sealed, and then cured at (20 ± 1) °C. The AEC-201 automatic machine was used to obtain the compressive strength of mortar at 1, 7, 14, and 28 days. The rate of load rate was 2.4 kN/s.

### 2.3. Autogenous Shrinkage

**Figure 2:**
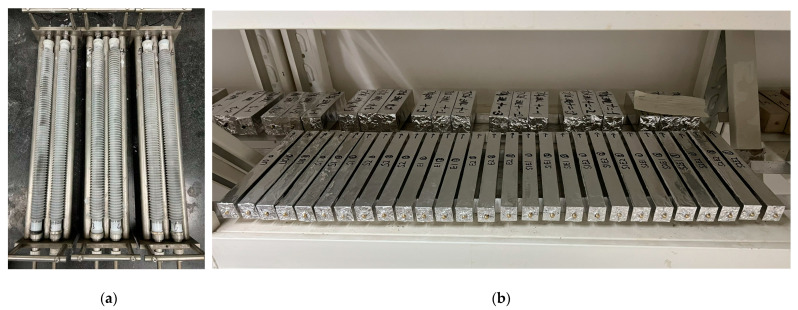
Samples in autogenous shrinkage test. (**a**) Specimens for early deformation (0–3 d) test; (**b**) Specimens for later deformation (3 d–28 d) test.

In order to accurately measure the deformation of the UHPC matrix in 28 days, the autogenous shrinkage at early ages (0–3d) was obtained according to ASTM C1698 [29]. The length of the samples was approximately 400 mm. The specimens were placed in a plastic bellows, which were immersed in a temperature-controlled glycol bath at (20 ± 0.1) °C. Two samples were simultaneously tested in each batch of the UHPC matrix, with a measuring accuracy of ±5 μm/m. Continuous linear measurements were carried out directly after casting, and the results were zeroed at the final setting time. The samples are presented in Figure 2a. The autogenous shrinkage at longer ages was tested according to Chinese Standard GB/T 29417-2012 [30]. The mold size of the test piece was 25 × 25 × 280 mm^3^. The sealed samples were cured at (20 ± 1) °C. From the start of 1.3d, the length change in the test pieces was monitored for 28 days. Three samples were tested simultaneously in each batch of the UHPC matrix. The samples were presented in Figure 2b.

We used different testing techniques at different stages for these reasons:

According to GB/T 29417-2012, the early data cannot be tested before the final set and demolding of sample. The early strength of the sample was low, and essential movement during testing might cause erroneous strain. Therefore, this method works better for obtaining long-term deformation of UHPC matrix.

In tests according to ASTM C1698, early deformation was measured immediately after mixing. Additionally, the data are more precise and continuous than those of GB/T 29417-2012. However, in the long-term measurement, ASTM C1698 usually exceeded the measurement range of LVDT probe. Therefore, ASTM C1698 was used to test for deformation in early ages.

### 2.4. Relative Humidity

To study the effect of internal humidity on the autogenous shrinkage of UHPC. RH was measured using the Rotronic Water Activity HC2-SH Sensor. The nominal accuracy (true degree) of the sensor was (±0.5%) RH. Before and after the measurement, the sensor was calibrated using three saturated salt solutions of CaCl_2_, KCl, and Na_2_CO_3_ to balance the relative humidity values between 75 and 98%. The samples and sensors were kept at (20 ± 0.2) °C. The UHPC matrix was crushed into small pieces after 24 h (the diameter was approximately 5 mm). Approximately 200 g of samples were inserted in the sealed measuring chamber, and data were collected every 10 min for 21 days.

### 2.5. Low-Field ^1^H Nuclear Magnetic Resonance

To obtain an accurate value of internal humidity and verify the homogeneity of humidity distribution during internal curing, Niumag MesoMR12-060V-1 instrument (Niumag Analytical Instrument Co., Ltd., Suzhou, China) was used for the ^1^H-nuclear magnetic resonance (NMR) test. The magnetic field strength was 0.3T, and magnetic field uniformity was less than 30 ppm. The magnetic field stability was less than 200 Hz/h, and the pulse frequency range of the radio frequency field was 1~30 MHz. The peak output of radio frequency transmission power was greater than 300 W, the maximum sampling bandwidth was 2000 kHz, and the sampling rate was 50 mHz. The minimum echo time was 60 μs. Gas temperature control technology was adopted, and the temperature control accuracy was (±0.3) °C. The RF coil diameter was 60 mm. Next, 20 g of fresh cement paste was poured into a chromatographic bottle with a height of 50 mm and an inner diameter of 20 mm and was sealed with a plastic film. The water signal intensity distribution on the vertical height axis of the cylindrical sample was obtained via one-dimensional frequency encoding (GR-HSE series).

### 2.6. Scanning Electron Microscopy with Energy-Dispersive X-ray Spectroscopy

To observe the distribution of CEA products, the UHPC matrix from 1 d, 7 d, 14 d, and 28 d was crushed into 1~2 mm particles and then immersed in isopropanol for 7 days to terminate hydration. Then, the sample was oven-dried at 60 °C for 48 h in a vacuum and then placed in a vacuum desiccator for storage. The microstructure was observed using Quanta 250 Filed Emission Scanning Electron Microscope (FEI, Hillsboro, OA, USA) coupled with AXS XFlash Detector 4030 EDS analyzer (maximum magnification: 1,000,000×, resolution: secondary electron (SE) imaging, maximum acceleration voltage: 30 kV, maximum pressure of sample chamber: 2600 Pa). Samples were observed at 15 kV accelerating voltage with a magnification of about 1000×.

### 2.7. TGA

To study the quantity of CEA products, the samples were ground into powder at 1, 7, 14, and 28 days. The samples were heated from room temperature to 900 °C at a rate of 10 °C/min in nitrogen atmosphere. The change in sample mass with the changing temperature was measured using NETZSCH-TG-209F3 thermogravimetric analyzer (NETZSCH-Gerätebau GmbH, Selb, Germany).

### 2.8. Pore Structure

In order to study the effects of SAP and CEA on the porosity and pore size distribution of the UHPC matrix. At the age of 1, 7, 14, and 28 days, hardened UHPC paste was crushed into 1~2 mm particles, and immediately rinsed and soaked with isopropanol. After the same dehydration treatment as the SEM samples, MicroActive AutoPore V 9600 mercury porosimeter (Micromeritics instrument Co., Ltd., Shanghai, China) was used to measure the pore size distribution and porosity of UHPC paste.

## 3. Results and Discussion

### 3.1. Compressive Strength

**Figure 3:**
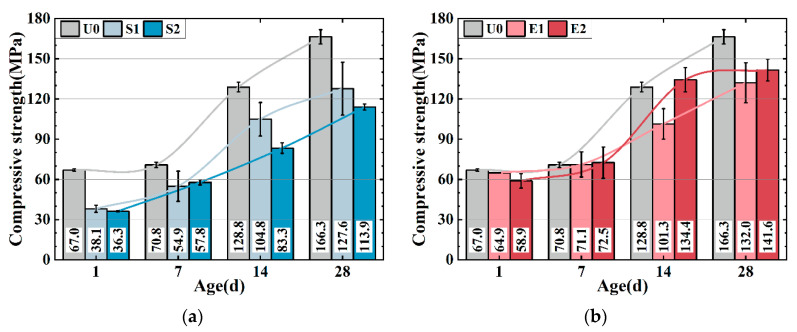

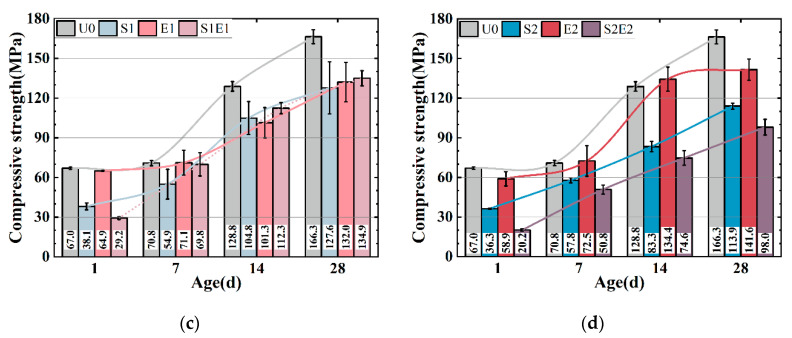
Compressive strength of the UHPC with SAP and CEA. (**a**)SAP series; (**b**) CEA series; (**c**) S1E1 series; (**d**) S2E2 series.

Figure 3a,b show the compressive strength of the UHPC matrix with only SAP (0.1 wt%) and CEA (2 wt%). The strength of the UHPC is weakened by SAP. S1 and S2 have 23.3% and 31.5% lower strengths compared to U0 at 28 d, which can be attributed to SAP desorption creating cavities in the matrix, reducing strength, and increasing actual w/b. S2 has a higher strength than S1 after 7 days, potentially because the introduction of more water may accelerate cement hydration, leading to a higher early strength development rate in S2. CEA decreases the UHPC matrix intensity as shown in Figure 3b. E1 and E2 show 20.6% and 14.4% lower strength compared to U0 after 28 d, while at 7 d and 14 d, the strength of E2 is relatively higher. Two possible explanations for the phenomenon can be proposed: firstly, the exothermic CEA reaction enhances cement hydration, while C-S-H gel densify UHPC matrix. Secondly, the CEA reaction produces more calcium hydroxide crystals, refining the pore structure of the UHPC. The formation of calcium hydroxide is discussed in detail in Section 3.5.

The compressive strength of S1E1 (Figure 3d) has a higher 28-day strength than those of groups with only SAP and CEA, indicating the enhancement of SAP on CEA hydration and matrix strength. However, high dosages of SAP and CEA in the UHPC matrix cause strength losses (S2E2 in Figure 3d). S2, E2, and S2E2 reduced 28-day strength by 31.5%, 14.9%, and 41.1%. The strength loss induced by SAP defects increased when dosage increased, culminating in strength loss.

### 3.2. Autogenous Shrinkage

#### 3.2.1. Autogenous Shrinkage at Early Ages

**Figure 4:**
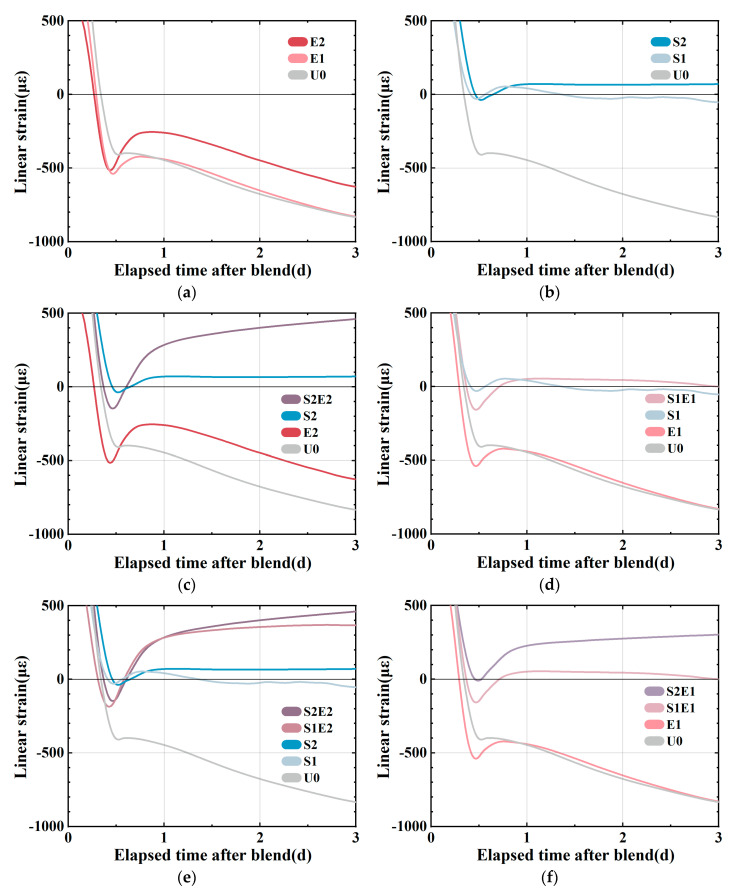
Autogenous shrinkage of the UHPC matrix before 3d (final setting time, 3d). (**a**) Deformation of matrix with CEA. (**b**) Deformation of matrix with SAP. (**c**) Deformation of S2E2. (**d**) Deformation of S1E1. (**e**) Effects of CEA in curing systems. (**f**) Effects of curing water in matrix with CEA.

As shown in Figure 4a, E1 and E2 still maintain a rapid shrinkage for a period of time after the final setting. In UHPC with EA, this quick shrinkage trend usually ends at around 0.5d [8]. At this stage, the generating capillary pressure drives the high deformation of the material since the rigid structure is rather weak. In Figure 4b, S1 and S2 are shown to expand slightly after the final setting [12] as a result of the combined effect of the expansion caused by hydration heat and the expansion of SAP water absorption. SAP releases water to compensate for chemical shrinkage and plays an internal curing role, so the shrinkage curve remains stable. The shrinkages of the S1E1 and S2E2 experimental groups (Figure 4c,d) are smaller than those of SAP and CEA alone after 1 day [8], and S2E2 continues to expand. Figure 4e shows the linear change between samples with and without CEA in the SAP curing system. In the absence of CEA, it is evident that increasing SAP content can lower or eliminate early shrinkage. At this stage, the over-expansion force may be obtained in a mixed system by adding CEA. A comparison of different levels of internal curing is shown in Figure 4f. The length change in 1 wt%CEA samples shows that a higher SAP content creates more expansion of the CEA samples. On the one hand, SAP releases water to mitigate shrinkage. On the other hand, CEA hydration products make up for shrinkage in different humidity conditions. Although the investigation of temperature strain is beyond the scope of this study, it is interesting to consider that SAP and CEA have opposing effects on hydration heat. CEA is shown to increase the rate of heat release during hydration, whereas SAP is shown to decrease it.

#### 3.2.2. Autogenous Shrinkage at Longer Ages

**Figure 5:**
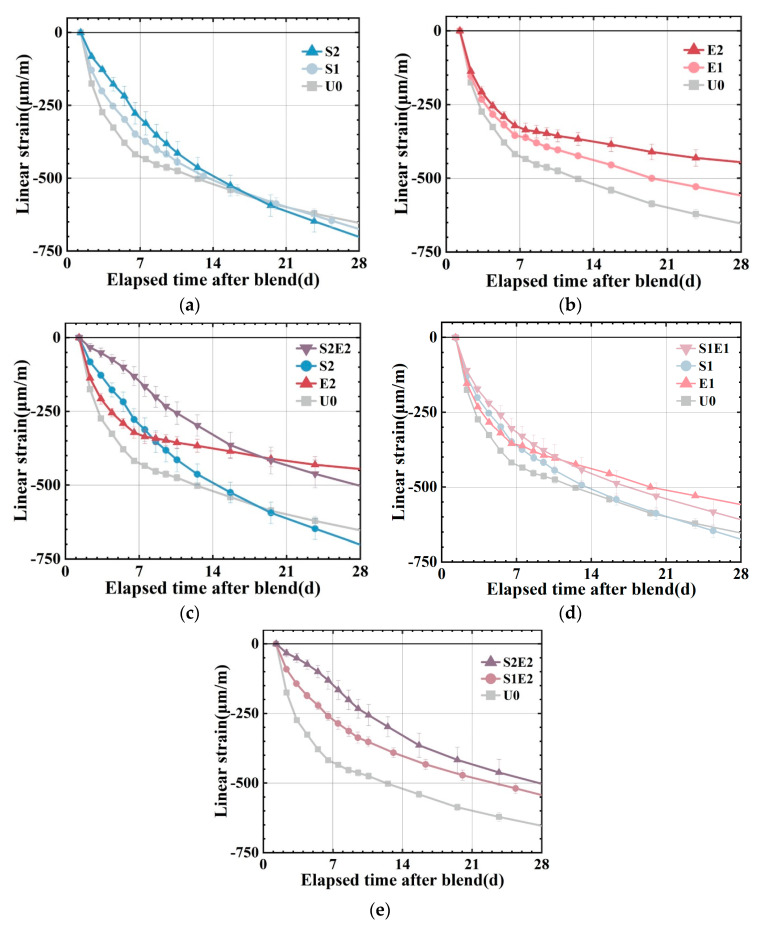
Autogenous shrinkage of UHPC matrix (1.3–28 d). (**a**) Deformation of matrix with SAP. (**b**) Deformation of matrix with CEA. (**c**) Deformation of S2E2. (**d**) Deformation of S1E1. (**e**) Different curing level in matrix with hybrid use of CEA and SAP.

The autogenous shrinkages at higher ages are presented in Figure 5a–d. U0, S1, and S2 have linear shrinkages of 430 με, 350 με, and 295 με at 7 d. S1 and S2 shrank 19% and 31% less than at U0. SAP content greatly reduced the early shrinkage of the UHPC matrix. However, SAP subsequently caused a higher shrinkage at about 20 d, which was also observed in ordinary mortar [25]. An overall shrinkage pattern of fast shrinkage in 7 days followed by a slowdown can be observed in U0, E1, and E2. As a comparison to the SAP series group, the CEA series group demonstrated a more consistent and long-lasting shrinkage compensatory effect. In S2E2, the combination of SAP and CEA lowers the early autogenous shrinkage of UHPC substantially. The shrinkage reduction effect of S2E2 after compound blending is 65%, which is significantly greater than the 31% reduction effect of S2 and the 26% reduction effect of E2, and the shrinkage rate is more stable and uniform in comparison to the reference and E2. At 28 d, the linear shrinkage of the S2E2 is larger than that of the E2. In S1E1, the shrinkage is lower than that of the reference (U0), and the 28 d strength is more than that of the SAP or CEA separately. It demonstrates the extensive benefits of a proper mix design in terms of strength and shrinkage control.

The 7 d relative expansion is compared with the 14 d relative expansion (reduction in shrinkage of the sample compared to the reference), as shown in Table 5, demonstrating the impact of varying degrees of internal curing on the effect of CEA. When SAP is 0.1 wt%, S1E2 expands by 160 με at 7 d relative to the reference, decreasing the microstrain of the reference by 37%. The relative expansion value of S2E2 7 d is 280 με when the SAP content is increased to 0.2 wt%, which is 65% less than the autogenous shrinkage of the reference group. It demonstrates that the higher the amount of internal curing water, the greater the potential for CEA expansion.

Based on the measured autogenous shrinkage of different samples, the interaction between SAP and CEA on autogenous shrinkage can be summarized as follows:

At an early stage, the early growth potential of CEA can be effectively promoted by the internal curing water and dramatically expands when the internal curing level increases.

At a later stage, the relative expansion value does not continue to increase as fast as when only CEA is added. The contribution of SAP in promoting CEA hydration focuses mostly on the initial stage. These vanishing values of linear expansion may be linked to the pores generated by the water release and SAP collapse. This hypothesis is supported by the pore structure and SEM images in Section 3.4.

This is also consistent with the time window in which UHPC rapidly contracts during the early stage and slowly during the late stage, generating a linear and flat shrinkage curve of S2E2.

### 3.3. Internal Relative Humidity and Water Distribution

**Figure 6:**
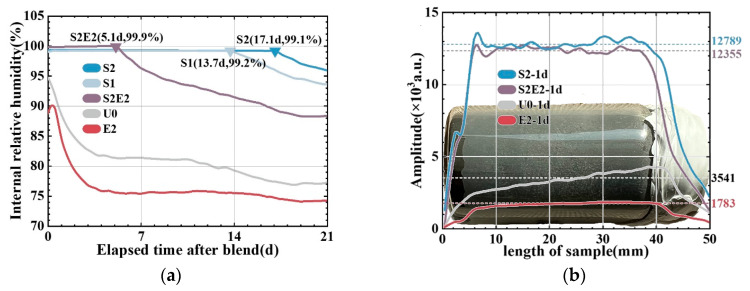
Relative humidity change in hardened UHPC matrix. (**a**) RH change with time. (**b**) Water distribution in the 1 d matrix.

The adsorption and desorption of water by SAP will alter the space–time distribution of water in the matrix. Figure 6 shows the effects of the composite of two functional materials on the internal relative humidity of the matrix. At the probe-balancing stage, a peculiar trend of rising humidity was observed, as shown on the left side of the humidity curve. During this period, the moisture released by the newly smashed test pieces gradually reach equilibrium to relative humidity of the air in the testing container. The RH of the reference is 94.5% after the humidity equilibrium (reaching its maximum) and is in a state of continuous decline. For the first 17 days, the RH of S2 remains above 99%, suggesting that the system is still in a water-saturated state. Notably, the high relative humidity is difficult to accurately monitor using an RH probe, and SAP contains residual water [31]. CEA severely lowered the initial internal relative humidity, leaving the system drier and durative lack of water, see the result of E2. After the CEA consumes the water in SAP, a rapid descent in internal humidity can also be seen in S2E2, in which a platform period of water saturation only lasts approximately five days. CEA significantly increases the water consumption rate, showing that an endogenous water supply is the most important aspect for ensuring the expansion of CEA.

Early RH data (before 1 d) are unreliable due to their balancing time, and no distribution information can be obtained within the specimen. Therefore, the early humidity distribution of the UHPC matrix is investigated using ^1^H-NMR and one-dimensional linear coding to quantitatively calculate the improvement rate of the curing effect on the UHPC matrix at the age of 1 day.

Figure 6b shows the moisture accumulation signal cross-section of the one-dimensional cylindrical sample at 24 h. SAP increases the internal free water content by 261% (in S2) compared to the reference, confirming the finding of the humidity probe test. Furthermore, in the unit volume matrix, 2 wt% CEA consumes 1758 units more water than U0 when there is no internal water supply. It only consumes 434 units more water than S2 when there is an internal water supply. In a lower curing level, CEA causes the matrix to consume higher amounts of water, which indicates that CEA has a higher hydration competitiveness than cement clinker. It also means that sufficient curing water in SAP can not only accelerate the hydration of CEA but also avoid the negative effect of CEA on reducing the hydration degree of cement due to hydration competition in low RH [18].

### 3.4. Pore Structure

**Figure 7:**
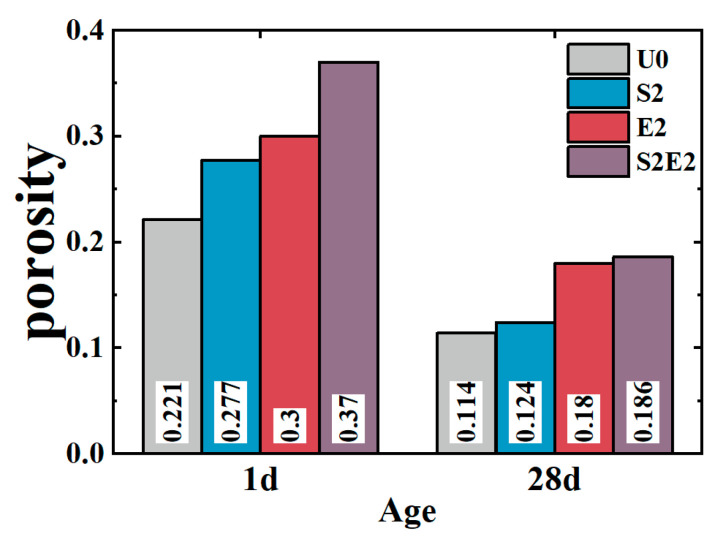
Total porosity of UHPC matrix.

**Figure 8:**
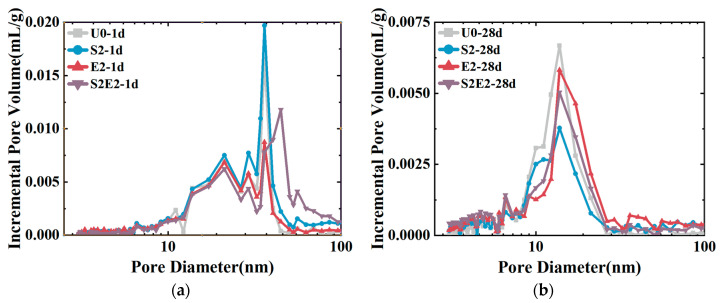
Incremental pore volume distribution: (**a**) 1 d. (**b**) 28 d.

As shown in Figure 7, at 1 d, the total cumulative porosity values of U0, S2, E2, and S2E2 were 22.1%, 27.7%, 30%, and 37.0%, respectively. At 28 d, the porosity values of U0, S2, E2, and S2E2 were reduced to 11.4%, 12.0%, 18.0%, and 18.8%, respectively, as a result of the hydration products filling the pores. CEA and SAP both raise the overall porosity of the UHPC matrix. In the presence of a liquid phase, the inner capillary liquid surface of the matrix creates shrinkage stress. When the capillary pore size decreases, the shrinkage stress rapidly increases. There is a clear proportional association between the volume ratio of pores at 5–50 nm and cement paste shrinkage [32]. Additionally, the micromechanical properties of C(-A)-S-H compacts are dominated by porosity [33]. The integration of CEA reduces the porosity within the range of 5–50 nm aperture and increases the porosity within the range of 50 nm aperture, as shown in Figure 8 and Table 6. Consequently, the addition of CEA can increase the pore size of cement paste and decrease the number of pores in the range of 5–50 nm at 1 d, which is directly associated with shrinkage. CEA in S2E2 inherits this feature at an early age.

At the early stage, SAP inclusion has little influence on the pore structure of the UHPC matrix. With the continual formation of hydration products, the 28 d microporosity of UHPC matrix under internal curing conditions is considerably reduced, resulting in a more compact microstructure. This phenomenon also occurs in S2E2.

### 3.5. Formation of Calcium Hydroxide (CH)

**Figure 9:**
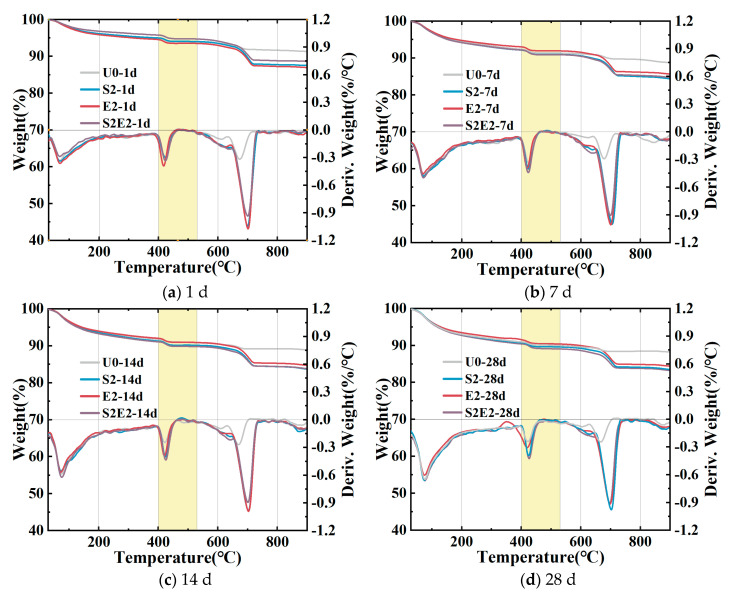
TG curves of U0, S2, E2, and S2E2. (**a**) 1 d; (**b**) 7 d; (**c**) 14 d; (**d**) 28 d.

The amount of CH formed in different samples (based on the TGA curve in Figure 9) via weight loss at 400–530 °C [32,34,35] is shown in Table 7. Firstly, adding CEA or SAP alone will increase the CH content in the UHPC at various time periods. Secondly, the amount of CH in E2 is highest after 1 day, and thus lowers the amount of micropores. At 1 day, the content of CH in the E2 is greater than that of the S2E2, demonstrating that the rate of CH formation is accelerated when the system lacks water. This will result in a quicker increase in the alkalinity of the pore solution, which is consistent with the early strong impact of CEA discussed in Section 3.1. Thirdly, in the DTG curve of UHPC matrix cured in 0.2 wt% SAP, a rise in CH is noticed at 430 °C, showing that SAP might cause the UHPC to form CH crystalline phase. At 28 d, the CH levels are at their maximum in S2E2. This contradicts the degree of S2E2 deformation, as shown in Figure 5c.

**Figure 10:**
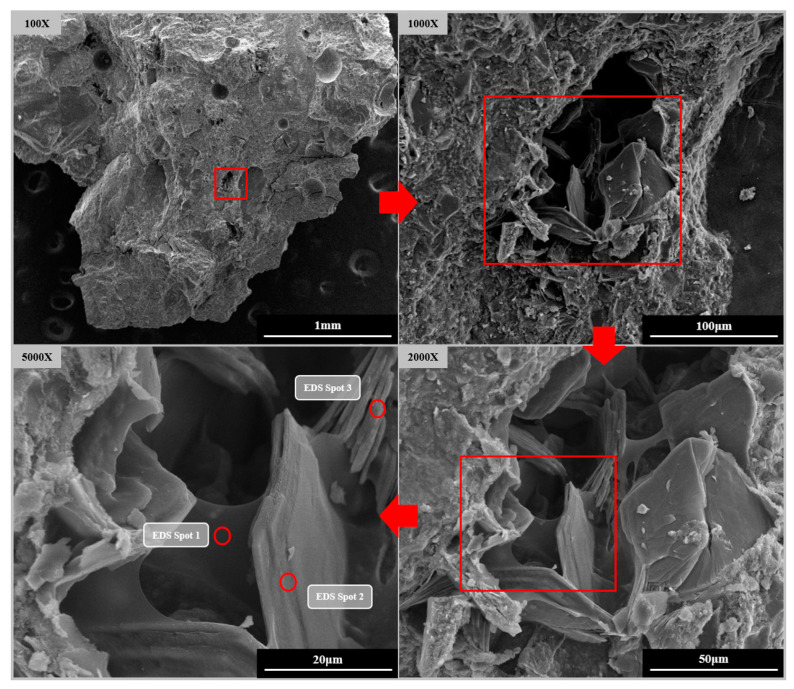
Pores formed by SAP in S2E2.

Figure 10 illustrates the microstructure of S2E2 at 7 days. In contrast to the typical UHPC matrix, in which the microstructure has a high density, the defect induced by SAP water loss is visible on the composite group picture. Table 8 displays the element distribution generated from EDS measurements at each position in Figure 10. It has been deduced that the film-like material in the micrograph that possesses a delicate shine is organic, in other words, it is the residue left behind when SAP loses its water content. These residues envelope hexagonal CH crystals of 50–100 μm in size. Notably, this phenomenon is not found in the U0, E2, or S2.

A large quantity of CH crystals forms in SAP pores with sizes of 50~300 μm, which may be caused by the following: (1) The emptied SAP provides enough space for the generation of CH crystals. (2) Especially when compared to the dry matrix, the SAP contains a greater quantity of water, which supplies reactants for CEA hydration products. (3) The decreased microporosity of S2 in comparison to the reference (U0) suggests that SAP enhances the degree of hydration to make the microstructure of the UHPC matrix denser, hence driving free CH to crystallize at the hole wall.

In any case, these CH crystals have much lower expansion stress, which explains why vast quantities of CH are produced during the hybrid use of SAP and CEA without a comparable decrease in shrinkage.

Moreover, according to Table 9, the accumulation of 50~300 μm pore volume demonstrates that the macropore porosity in this range is not reduced by CEA. Compared to S2 and S2E2, SAP shows no signs of the refinement of macropores.

## 4. Conclusions

Based on the experimental results and discussions above, the following conclusions can be drawn:

(1) The reasonable hybrid use of SAP and CEA can have higher strength and lower shrinkage compared with the single use of SAP or CEA. SAP appears to have a negative effect on long-term shrinkage reduction, whereas CEA can overcome this unfavorable effect. At 28 days, the autogenous shrinkage of S2E2 was 36% less than that of S2. The curing water in SAP can alter the CEA expansion process. At 28 days, 13% more calcium hydroxide was found in S2E2 than in E2. Hybrid use enhances the time window matching between material expansion and specimen shrinkage, which provides a practical measure to mitigate autogenous shrinkage in the UHPC matrix.
(2) With a lower curing level, CEA causes the matrix to consume higher amounts of water, which shows that CEA has a higher hydration competitiveness than cement clinker. Sufficient curing water in SAP can not only accelerate the hydration of CEA but also avoid the negative effect of CEA on reducing the hydration degree of cement due to hydration competition in a low-humidity environment.
(3) At 28 d, the CH (CEA products) was at a very high level (about 3.9 wt%) in S2E2 but had a high deformation. This phenomenon in hybrid use samples can be explained by the suppressed crystallization pressure of growing CH in the extra space provided by the emptied SAP. This new finding helps to clarify the mechanisms via the combined use of SAP and CEA in mitigation shrinkage in UHPC.

## Figures and Tables

**Table 1:** Chemical composition of the raw materials.

Raw Material (wt%)	CaO	SiO_2_	Al_2_O_3_	Fe_2_O_3_	K_2_O	MgO	Na_2_O	SO_3_	TiO_2_	ZrO_2_
P·Ⅱ52.5 cement	63.704	19.345	3.991	3.085	0.522	1.57	0.125	2.798	0.261	0
SCMs	14.52	29.31	40.52	1.57	0.81	4.27	0.9	1.11	2.57	3.32

**Table 2:** Phase composition of CEA (wt%).

CaO	CaSO_4_	CaCO_3_	Ca(OH)_2_	C4A3S	CaMg(CO_3_)_2_	CaSO_4_∙2H_2_O	SiO_2_
35.26	34.09	1.33	0.45	27.13	0.39	0.16	1.19

**Table 3:** Particle size distribution of SAP (wt%).

<100 μm	100–125 μm	125–180 μm	180–300 μm	>300 μm
44	20.1	20.4	15.3	0.2

**Table 4:** Mix proportion of UHPC matrix (kg/m^3^).

Samples	Cementitious Material					Water	SuperPlasticizer	Sand	SAP	Additional Water
	Cement	Silica Fume	FA	Ultra-Fine Calcium Carbonate	CEA					
U0	802	147	107	107	-	191.3	17.43	930	-	-
S1	802	147	107	107	-	191.3	17.43	930	1.16	23.3
S2	802	147	107	107	-	191.3	17.43	930	2.32	46.5
E1	794	145	105	105	11.62	191.3	17.43	930	-	-
E2	786	144	104	104	23.24	191.3	17.43	930	-	-
S1E1	794	145	105	105	11.62	191.3	17.43	930	1.16	23.3
S1E2	786	144	104	104	23.24	191.3	17.43	930	1.16	23.3
S2E1	794	145	105	105	11.62	191.3	17.43	930	2.32	46.5
S2E2	786	144	104	104	23.24	191.3	17.43	930	2.32	46.5

**Table 5:** Reduction in shrinkage of SAP and CEA in different periods.

Samples	Shrinkage Reduction at 7 d/με	Shrinkage Reduction at 14 d/με
S1E2	160	120
S2E2	280	190

**Table 6:** Accumulative volume of 5–50 nm pore (mL/g).

Age	U0	S2	E2	S2E2
1 d	0.069	0.086	0.058	0.070
28 d	0.032	0.025	0.031	0.029

**Table 7:** Calcium hydroxide amount in the UHPC matrix (wt%).

Ages	U0	S2	E2	S2E2
1 d	2.91	2.96	3.29	3.13
7 d	3.15	3.75	3.38	4.05
14 d	2.43	3.62	3.38	3.92
28 d	2.47	3.37	3.43	3.88

**Table 8:** Elements at each point of S2E2 (wt%).

EDS Spot	C	O	Si	Ca	Else
Spot 1	7.46	28.10	3.13	61.31	0.00
Spot 2	7.37	48.67	0.00	40.24	3.72
Spot 3	9.67	42.31	1.45	41.41	5.16

**Table 9:** 50~300 μm pore volume accumulation (mL/g).

Age	U0	S2	S2E2
1 d	0.041	0.037	0.144
7 d	0.025	0.026	0.105
14 d	0.020	0.017	0.047
28 d	0.020	0.017	0.045

## Data Availability

No new data were created or analyzed in this study. Data sharing is not applicable to this article.

## References

[1] Ali I., Burakov A.E., Melezhik A.V., Babkin A.V., Burakova I.V., Neskomornaya M.E.A., Galunin E.V., Tkachev A.G., Kuznetsov D.V. (2019). Removal of Copper(II) and Zinc(II) Ions in Water on a Newly Synthesized Polyhydroquinone/Graphene Nanocomposite Material: Kinetics, Thermodynamics and Mechanism. ChemistrySelect.

[2] Basheer A.A. (2020). Advances in the smart materials applications in the aerospace industries. Aircr. Eng. Aerosp. Technol..

[3] Ali I., Kucherova A., Memetov N., Pasko T., Ovchinnikov K., Pershin V., Kuznetsov D., Galunin E., Grachev V., Tkachev A. (2019). Advances in carbon nanomaterials as lubricants modifiers. J. Mol. Liq..

[4] Shi C., Wu Z., Xiao J., Wang D., Huang Z., Fang Z. (2015). A review on ultra high performance concrete: Part I. Raw materials and mixture design. Constr. Build. Mater..

[5] Golewski G.L., Szostak B. (2022). Strength and microstructure of composites with cement matrixes modified by fly ash and active seeds of C-S-H phase. Struct. Eng. Mech..

[6] Cuesta A., Morales-Cantero A., De la Torre A.G., Aranda M.A.G. (2023). Recent Advances in C-S-H Nucleation Seeding for Improving Cement Performances. Materials.

[7] Yuan B., Wang H., Jin D., Chen W. (2023). C-S-H Seeds Accelerate Early Age Hydration of Carbonate-Activated Slag and the Underlying Mechanism. Materials.

[8] Liu L., Fang Z., Huang Z., Wu Y. (2022). Solving shrinkage problem of ultra-high-performance concrete by a combined use of expansive agent, super absorbent polymer, and shrinkage-reducing agent. Compos. B Eng..

[9] Yang L., Shi C., Wu Z. (2019). Mitigation techniques for autogenous shrinkage of ultra-high-performance concrete—A review. Compos. Part B Eng..

[10] Wu L., Farzadnia N., Shi C., Zhang Z., Wang H. (2017). Autogenous shrinkage of high performance concrete: A review. Constr. Build. Mater..

[11] Mechtcherine V., Wyrzykowski M., Schröfl C., Snoeck D., Lura P., De Belie N., Mignon A., Van Vlierberghe S., Klemm A.J., Almeida F.C. (2021). Application of super absorbent polymers (SAP) in concrete construction—Update of RILEM state-of-the-art report. Mater. Struct./Mater. Constr..

[12] Piérard J., Pollet V., Cauberg N. (2006). Mitigating autogenous shrinkage in hpc by internal curing using superabsorbent polymers. Proceedings of the International RILEM Conference on Volume Changes of Hardening Concrete: Testing and Mitigation.

[13] Zhao H., Li J., Sun G., Jiang J., Xuan W., Chen Y., Tian Q., Liu J. (2020). Effects of pre-soaked zeolite and CaO-based expansive agent on mechanical properties and autogenous deformation of early-age concrete. Constr. Build. Mater..

[14] Mo L., Fang J., Huang B., Wang A., Deng M. (2019). Combined effects of biochar and MgO expansive additive on the autogenous shrinkage, internal relative humidity and compressive strength of cement pastes. Constr. Build. Mater..

[15] Liu J., Farzadnia N., Shi C., Ma X. (2019). Shrinkage and strength development of UHSC incorporating a hybrid system of SAP and SRA. Cem. Concr. Compos..

[16] Chatterji S. (1995). Mechanism of expansion of concrete due to the presence of dead-burnt Cao and Mgo. Cem. Concr. Res..

[17] Corinaldesi V., Nardinocchi A., Donnini J. (2015). The influence of expansive agent on the performance of fibre reinforced cement-based composites. Constr. Build. Mater..

[18] Snoeck D., Pel L., De Belie N. (2017). The water kinetics of superabsorbent polymers during cement hydration and internal curing visualized and studied by NMR. Sci. Rep..

[19] Jensen O.M., Hansen P.F. (2001). Water-entrained cement-based materials I. Principles and theoretical background. Cem. Concr. Res..

[20] Jensen O.M., Hansen P.F. (2002). Water-entrained cement-based materials II. Experimental observations. Cem. Concr. Res..

[21] Li M., Liu J., Tian Q., Wang Y., Xu W. (2017). Efficacy of internal curing combined with expansive agent in mitigating shrinkage deformation of concrete under variable temperature condition. Constr. Build. Mater..

[22] Gwon S., Ahn E., Shin M. (2019). Self-healing of modified sulfur composites with calcium sulfoaluminate cement and superabsorbent polymer. Compos. B Eng..

[23] Zhao H., Jiang K., Di Y., Xu W., Li W., Tian Q., Liu J. (2019). Effects of curing temperature and superabsorbent polymers on hydration of early-age cement paste containing a CaO-based expansive additive. Mater. Struct./Mater. Constr..

[24] Wyrzykowski M., Terrasi G., Lura P. (2018). Expansive high-performance concrete for chemical-prestress applications. Cem. Concr. Res..

[25] Zhang M., Aba M., Sakoi Y., Tsukinaga Y., Shimomukai K., Kuang Y. (2021). Synergetic Effect of Expansive Agent (KEA) and Superabsorbent Polymers (SAP) on the Shrinkage, Strength and Pore Structures of Mortars. J. Adv. Concr. Technol..

[26] Ávalos-Rendón T.L., Mendoza C.J. (2017). Study on the Expansion of a Cement-Based System Containing Sap Polymer and Supplementary Cementing Materials. Mater. Sci. Appl..

[27] Zhang S., Lu Z., Li Y., Ang Y., Zhang K. (2021). A Method for Internal Curing Water Calculation of Concrete with Super Absorbent Polymer. Adv. Civ. Eng..

[28] (2021). Test Method for Strength of Cement Mortar (ISO).

[29] (2014). Standard Test Method for Autogenous Strain of Cement Paste and Mortar.

[30] (2012). Test Method for Drying Shrinkage Cracking of Cement Mortar and Concrete.

[31] Schlitter J.L., Bentz D.P., Weiss W.J. (2013). Quantifying Stress Development and Remaining Stress Capacity in Restrained, Internally Cured Mortars. ACI Mater. J..

[32] Li Y., Bao J., Guo Y. (2010). The relationship between autogenous shrinkage and pore structure of cement paste with mineral admixtures. Constr. Build. Mater..

[33] Wang J., Hu Z., Chen Y., Huang J., Ma Y., Zhu W., Liu J. (2022). Effect of Ca/Si and Al/Si on micromechanical properties of C(-A)-S-H. Cem. Concr. Res..

[34] Justice J.M., Kurtis K.E. (2007). Influence of Metakaolin Surface Area on Properties of Cement-Based Materials. J. Mater. Civ. Eng..

[35] Poon C.-S., Lam L., Kou S.C., Wong Y.-L., Wong R. (2001). Rate of pozzolanic reaction of metakaolin in high-performance cement pastes. Cem. Concr. Res..

